# Molecular Discrimination of *Cynanchum wilfordii* and *Cynanchum auriculatum* by InDel Markers of Chloroplast DNA

**DOI:** 10.3390/molecules23061337

**Published:** 2018-06-01

**Authors:** Yonguk Kim, Hakjoon Choi, Jawon Shin, Ara Jo, Kyung-Eun Lee, Seung-Sik Cho, Yong-Pil Hwang, Chulyung Choi

**Affiliations:** 1 Jeonnam Institute of Natural Resources Research, Jangheung-gun, Jeollanam-do 59338, Korea; kyu9801@hanmail.net (Y.K.); ohchj12@naver.com (H.C.); sjo8127@naver.com (J.S.); joara9153@naver.com (A.J.); goodlky4617@naver.com (K.-E.L.); 2 Department of Pharmacy, College of Pharmacy, Mokpo National University, Muan-gun, Jeollanam-do 58554, Korea; siason1@naver.com; 3 Department of Pharmaceutical Engineering, International University of Korea, Jinju-si, Gyeongsangnam-do 52833, Korea; protoplast@hanmail.net

**Keywords:** InDel markers, *Cynanchum wilfordii* Radix, *Cynanchum auriculatum* Radix, chloroplast genome sequence, Korean pharmacopoeia

## Abstract

The tuber of *Cynanchum wilfordii* (*Baekshuoh* Radix in Korean) is an important medicinal herb in Korea and China; however, it is difficult to differentiate *C. wilfordii* from a related medicinal herb, *C. auriculatum* (*Baishouwu* Radix in Chinese)*.* We sought to develop a molecular method that could be used to distinguish between the tubers of *C. wilfordii* and *C. auriculatum*. We aligned the chloroplast genome sequences (available in the NCBI database) of the two species and identified three species-specific insertion and deletion (InDel) sites in the *trnQ-psbK*, *rps2-rpoC2*, and *psaJ-rpl33* intergenic spacer (IGS) regions. To confirm the presence of these three InDels and validate their use as markers, we designed three primer pairs to amplify the *trnQ-psbK*, *rps2-rpoC2*, and *psaJ-rpl33* IGS regions. Polymerase chain reaction (PCR) amplification of the *trnQ-psbK* IGS region yielded a 249 bp fragment for *C. wilfordii*, and 419 bp fragment for *C. auriculatum*, whereas the *rps2-rpoC2* IGS primers produced a 629 bp fragment from *C. wilfordii* and a 282 bp fragment from *C. auriculatum*. In the *psaJ-rpl33* IGS region, allele fragments of 342 and 360 bp in length were amplified from *C. wilfordii*, whereas 249 and 250 bp fragment were amplified from *C. auriculatum*. We propose these three InDel markers as a valuable, simple, and efficient tool for identifying these medicinal herbs and will thus reduce adulteration of these herbal materials in commercial markets.

## 1. Introduction

**Figure 1:**
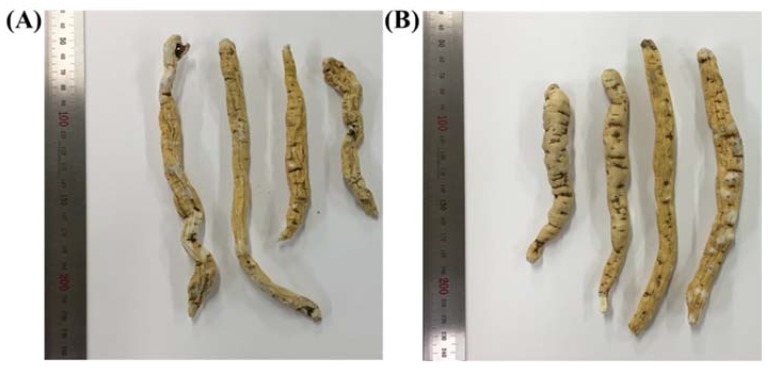
Commercial dried samples of *Cynanchum wilfordii* Radix (**A**) and *Cynanchum auriculatum* Radix (**B**).

*Cynanchum* (Apocynaceae) is a plant genus containing about 300 species. Among them, *C. wilfordii* (Maxim.) Hemsl. (*Baekshuoh* in Korean) and *C. auriculatum* Royle ex Wight (*Iyeobupiso* in Korean, *Baishouwu* in Chinese) are important traditional medicinal herbs in Korea and China [1,2]. Both species have active components in their tuber extracts that are considered effective for the prevention and treatment of various conditions, such as rheumatoid arthritis, geriatric diseases, atherosclerotic vascular diseases, and ischemia-induced diseases [3,4,5,6,7]. The name *C. wilfordii* Radix is frequently used for the tubers of both *C. wifordii* and *C. auriculatum*; however, only the dried tubers of *C. wilfordii* are stated in the Korean Herbal Pharmacopoeia as the origin plant of *C. wilfordii* (Maxim.) Hemsl. Because it is extremely difficult to morphologically distinguish between the tubers of the two species (Figure 1), and because *C. auriculatum* grows more rapidly and is more productive than *C. wilfordii*, the tuber of *C. auriculatum* has been used under the name *C. wilfordii* Radix as a functional food product.

Commercial products often contain a mix of tubers from *C. wilfordii* and *C. auriculatum,* which has created a problem in Korea. About 65% of *C. wilfordii* Radix products circulated in the market were found to contain added *C. auriculatum* Radix [8]. Therefore, recent studies have attempted to develop various molecular markers that can discriminate between the two species. Methods that have been used include random amplified polymorphic DNA (RAPD)-derived sequence-characterized amplified region (SCAR) markers [9], single nucleotide polymorphism (SNP) markers based on amplification-refractory mutation system–polymerase chain reaction (ARMS-PCR) and high resolution melting (HRM) analysis [10], SNP markers based on quantitative real-time PCR using SYBR green [11], and SNP markers based on multiplexed PCR [12]. Several of these markers have been approved for use by the Korean Food and Drug Administration (KFDA). In 2016, the complete chloroplast genomes of these two species were sequenced by de novo assembly using a small amount of whole genome sequencing data [13,14].

In this study, we tested for significant differences the complete chloroplast genome sequences of these two species. Our aim was to develop interspecies insertion and deletion (InDel) markers that can be used to distinguish between these two species and prevent indiscriminate mixing of these two Radix materials in the commercial product ‘Baekshuoh’.

## 2. Results and Discussion

**Figure 2:**
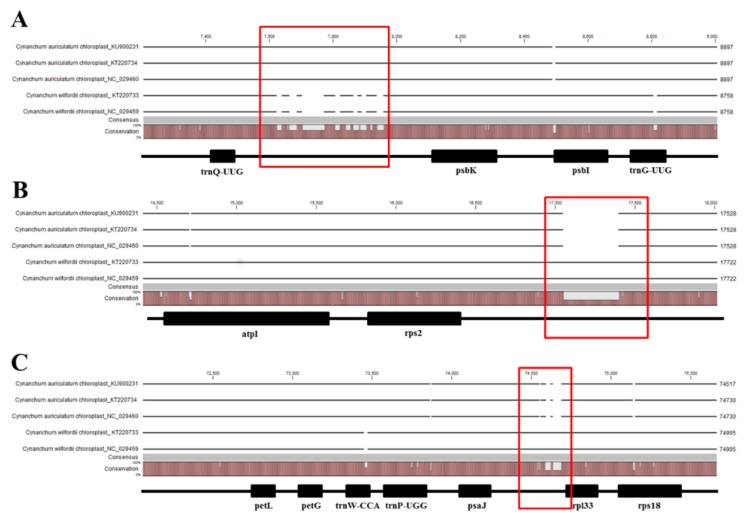
Multiple sequence alignment of five *C. wilfordii* and *C. auriculatum* chloroplast genomes derived from NCBI genbank using CLC Sequence Viewer 8 program. Multiple sequence alignments showed the three InDel regions with the possibility of candidate molecular markers. (**A**) InDels were observed in IGS region between *trnQ* and *psbK* genes. (**B**) InDel was observed in IGS region between *rps2* and *rpoC2* genes. (**C**) InDels were observed in IGS region between *psaJ* and *rpl33* genes. The red box denotes InDels.

Park et al., 2016 and Jang et al. 2016 derived the complete chloroplast genome sequences of *C. wilfordii* (Cw) and *C. auriculatum* (Ca), respectively [13,14], revealing total sequence lengths of 161,241 (*C. wilfordii*) and 160,203 or 160,840 bp (*C. auriculatum*; size difference due to allelic variants)—with a difference in genome size of 401 or 1038 bp between the two species. Based on multiple alignments of the complete chloroplast genome sequences of these two species in the National Center for Biotechnology Information’s (NCBI’s) GenBank public database, we identified three species-specific InDel regions including 8 InDels for Cw and 9 InDels for Ca among a total of 246 InDels. Expected amplicons could potentially be used to identify these two species. As these three InDel sequences were located in the *trnQ-psbK* IGS, *rps2-rpoC2* IGS, and *psaJ-rpl33* IGS regions of the chloroplast genome (Figure 2), we designed PCR primer pairs to amplify these regions by PCR (Table 1). Relative to Cw, Ca harbored the deletions of from 92–93 to 110–111 bp with nucleotide variation around the point of deletion in the *psaJ-rpl33* IGS region. Relative to Ca, Cw harbored an insertion of 347 bp and a deletion of 170 bp in the *rps2-rpoC2* IGS regions, respectively. We confirmed that these three InDel regions were consistently present by analyzing dried tuber samples from 10 individual plants of each species.

**Figure 3:**
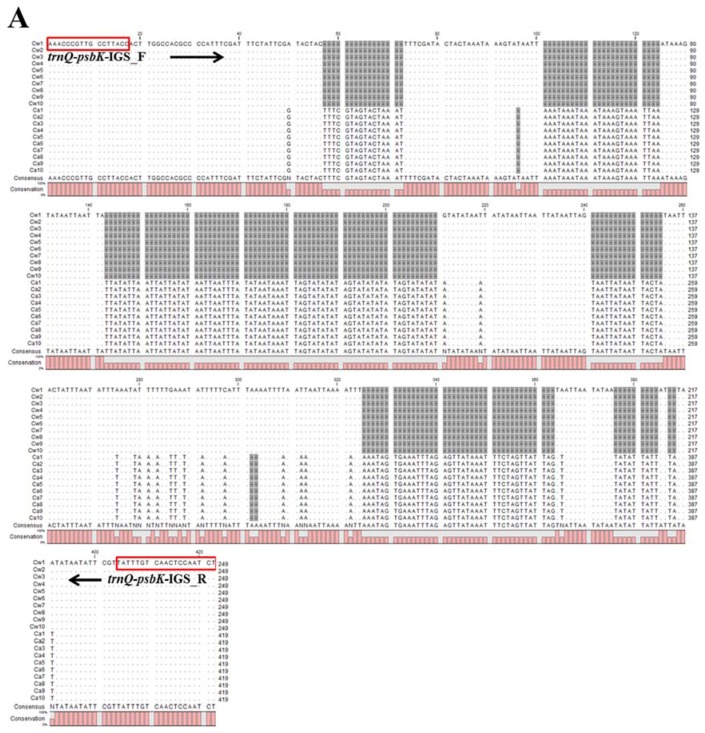

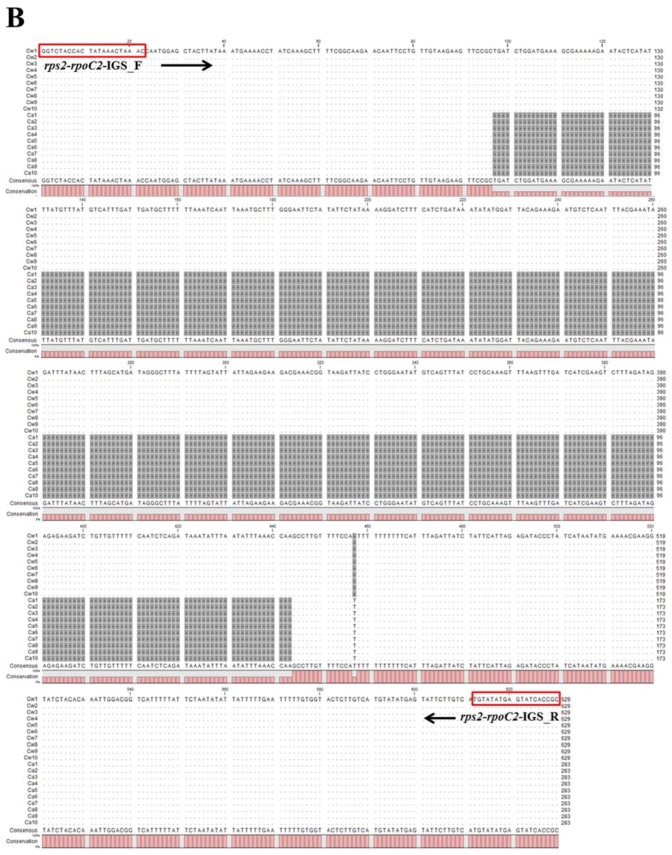

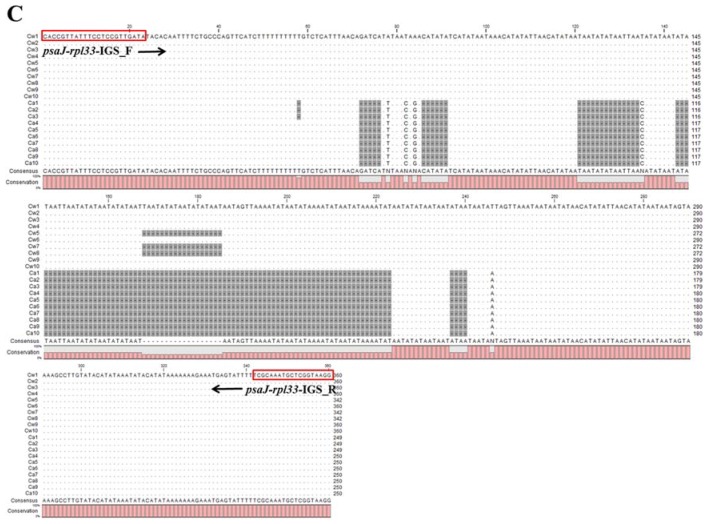
Sequence alignment of the *trnQ-psbK* (**A**), *rps2-rpoC2* (**B**), and *psaJ-rpl33* (**C**) intergenic space regions of *C. wilfordii* and *C. auriculatum* samples in this study. Arrows indicate the two designed primer sets (*trnQ-psbK*-IGS_F (forward) and R (reverse), *rps2-rpoC2*-IGS_F and R, and *psaJ-rpl33*-IGS_F and R); red boxes show the primer sequences.

Multiple sequence alignment (MSA) of the chloroplast *psaJ-rpl33* IGS region showed seven InDels, revealing two patterns with an approximately 15% polymorphism rate across all 20 samples (Figure 3C). Only one InDel pattern with a 100% polymorphism rate among all 20 samples was represented by MSA of the chloroplast *trnQ-psbK* and *rps2-rpoC2* IGS region, which included eight and two InDels, respectively (Figure 3A,B).

**Figure 4:**
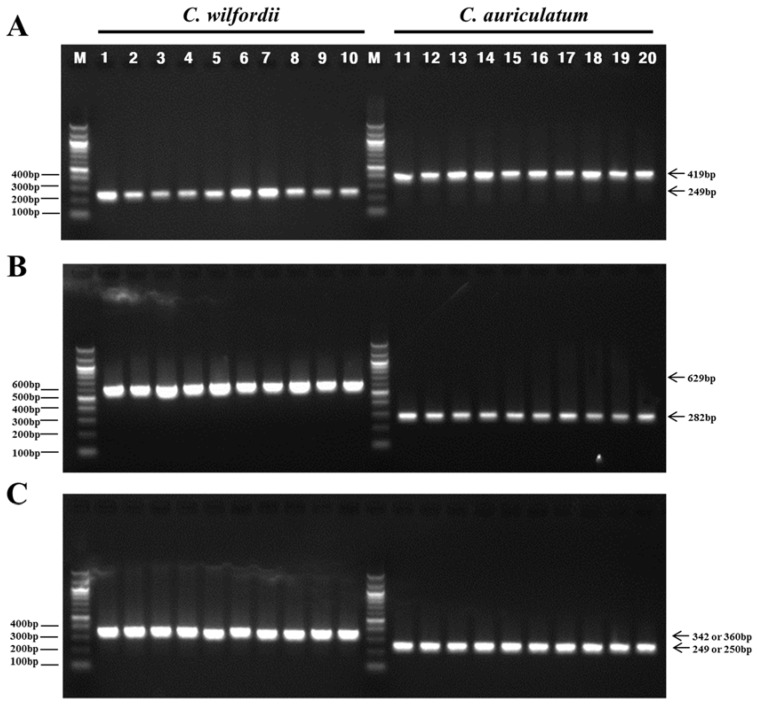
PCR analysis of *C. wilfordii* and *C. auriculatum* using primers designed to amplify the *trnQ-psbK* (**A**), *rps2-rpoC2* (**B**), and *psaJ-rpl33* (**C**) IGS regions. Lanes 1 to 10 are amplicons from *C. wilfordii* DNA; Lanes 11 to 20 are amplicons from *C. auriculatum* DNA; M, 100 bp DNA ladder.

PCR amplification using the *trnQ-psbK* IGS primers yielded species-specific amplicons of 249 bp (Cw) and 419 bp (Ca) (Figure 4A). The *rps2-rpoC2*-IGS primer pairs produced expected amplicons of 629 bp in the Cw samples and 282 bp in the Ca samples (Figure 4B), whereas the *psaJ-rpl33* IGS primers produced PCR amplicons of 342 or 360 bp in length in the Cw samples, and 249 or 250 bp in the Ca samples (Figure 4C).

**Figure 5:**
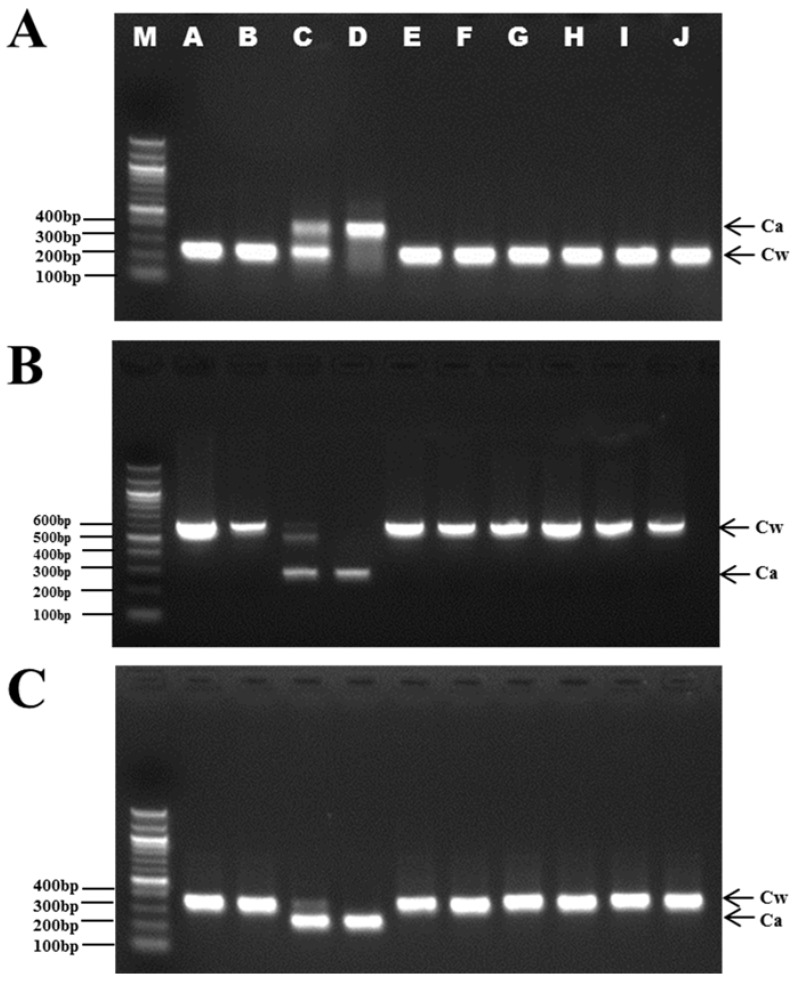
PCR identification of 10 commercial dried radix of Baekshuoh products. Lanes on 1.5% agarose gel: M: 100 bp DNA ladder; A–J: purchased commercial dried radix Baekshuoh products (see Table 2 for full details); 10 samples were amplified by PCR using three primer pairs; *trnQ-psbK*-IGS_F and R (**A**), *rps2-rpoC2*-IGS_F and R (**B**), and *psaJ-rpl33*-IGS_F and R (**C**).

Baekshuoh, a product of dried Radix of Cw, is regularly consumed as the herbal supplement in Korea. To validate the utility of PCR in commercial dried herbal materials, genomic DNA was extracted from tuber samples of 10 Baekshuoh products and amplified using the species-specific primers (Table 2). Almost all of the samples gave rise to clear two different band patterns consistent with the Cw and Ca, commonly used in Baekshuoh products (Figure 5). As shown in Figure 5, the band patterns revealed that A to J are Cw, while D was identified as being Ca. Interestingly, sample C showed that a double band pattern clearly detected both these two species as shown in Figure 5.

Optimized template DNA quantity isolated from dried tubers of Baekshuoh was 10–15 ng. Therefore, the gel image (Figure 5) clearly detected both Cw and Ca.

Our results demonstrated the specificity and sensitivity of these species-specific primers, which were used to distinguish between Cw and Ca.

The morphological similarity between *C. wilfordii* and *C. auriculatum* can result in adulteration, which ultimately leads to inferior quality of foods and herbal medicines. Studies have attempted to discriminate between the two species using molecular markers. Moon et al. (2010) established RAPD-derived SCAR markers for the simultaneous discrimination of *C. wilfordii*, *C. auriculatum*, and *Polygonum multiflorum* using multiplex PCR [9]. Kim et al. (2013) used a multiplexed PCR system to identify *C. wilfordii* Radix, *C. auriculatum* Radix, and *P. multiflorum* Radix using specific primer pairs derived from the chloroplast trnLF (tRNA-Leu) intron region [12]. Ryuk et al. (2014) developed a specific genetic marker for *C. wilfordii* using real-time PCR methods [11]. However, these methods are difficult to apply directly to herbal medicine materials and are unlikely to receive KFDA approval, because they have limited accuracy and reproducibility in their ability to discriminate between *C. wilfordii* and *C. auriculatum*. Now that the complete chloroplast genome has been sequenced for both species, we have better tools with which to identify these species, such as DNA barcoding and simple PCR methods. The results of our study indicate that it is possible to identify *C. wilfordii* and *C. auriculatum* using markers in the chloroplast *trnQ-psbK* IGS, *rps2-rpoC2* IGS, and *psaJ-rpl33* IGS regions. Compared with other molecular approaches, we predict this method will be convenient, efficient, and reliable for differentiating between *C. wilfordii* and *C. auriculatum* Radix materials in the pharmaceutical market.

## 3. Materials and Methods

### 3.1. InDels in Multiple Alignments of Chloroplast Genome Sequences

The InDel markers used in this study were identified by comparing the chloroplast genome sequences of *C. wilfordii* (GenBank accession numbers NC029459 and KT220733) and *C. auriculatum* (GenBank accession numbers NC029460, KU900231, and KT220734) available in the National Center for Biotechnology Information (NCBI) database (Table 3). These chloroplast genome sequences were aligned by using Clustal Omega tool (EMBL-EBI, www.ebi.ac.uk) [15], CLC sequence viewer version 8.0, and analyzed by using MEGA version 7.0 [16]. From a total of 246 InDel patterns identified in silico, three InDel regions were (each with insertions or deletions of >100 bp) used in this study to validate their ability to identify these two species. Primers were based on the sequences of these three InDel markers.

The designed InDel primer pairs were checked for target specificity in silico using Primer-BLAST (https://www.ncbi.nlm.nih.gov/tools/primer-blast/) against the NCBI non-redundant (nr) database, and showed absence of predicted cross reactivity with non-target templates. The selected InDel markers were designed using the Integrated DNA Technologies (IDT, Coralville, IA, USA) PrimerQuest tool interface (www.idtdna.com/Primerquest, IDT) [17]. InDel primer pairs were synthesized by Cosmo Genetech (Daejeon, Korea).

### 3.2. Sampling and Genomic DNA Extraction

Twenty dried radix samples (10 *C. wilfordii* and 10 *C. auriculatum*) were collected from verified market sources in Jecheon and Yeongju, Korea. Dried tubers of Baekshuoh products were purchased at local markets (Table 2). Species identification was performed by Herbal Medicine Division, National Institute of Food and Drug Safety Evaluation, Korea. Total genomic DNA was extracted from 0.3 g dried radix samples using a Plant DNeasy Extraction Kit (Qiagen, Hilden, Germany), following the manufacturer’s protocol. The quantity and quality of the extracted DNA were determined using a Nanodro ND-1000 (Thermo Fisher Scientific, Wilmington, DE, USA), after which the DNA was diluted to approximately 20–30 ng∙μL^−1^ for PCR amplification and stored at −20 °C until required.

### 3.3. PCR Amplification and DNA Sequencing

To amplify InDel regions, 30 ng of genomic DNA was used in a 50 μL total reaction volume that contained 10 pmols of each primer, 4 μL dNTP mixture (2.5 mM), 5 Units of Ex. Taq polymerase and 5 μL 10X Ex. Taq buffer with Mg^2+^ (20 mM) (TaKaRa, Kyoto, Japan). PCR amplification was carried out under the following conditions: initial denaturation at 95 °C for 5 min; 40 cycles of denaturation at 95 °C for 30 s, annealing at 55 °C for 30 s, and extension at 72 °C for 2 min; ending with an extension at 72 °C for 5 min. The resulting products were separated by 1.5% agarose gel electrophoresis, and the target DNA was extracted and purified using a QIAquick Gel Extraction Kit (Qiagen). The purified PCR products were introduced into a TA cloning vector using the Topcloner^TM^ TA Kit (Enzynomics, Daejeon, Korea), and the ligation products were transformed into *Escherichia coli* DH5-α competent cells. The recombinant plasmids were purified using a Plasmid Mini Kit (Qiagen) and sequenced at Cosmo Genetech (Seoul, Korea) using the Applied Biosystems 3730 DNA Analyzer (Thermo Fisher Scientific Corp., Waltham, MA, USA). Three colonies were sequenced for each DNA fragment, providing three replicates.

## Figures and Tables

**Table 1:** InDel markers used in this study.

Locus	Nucleotide Sequences (5′→3′)	Location (nt)	Expected PCR Product Size (bp)	
Primer Name			*C. wilfordii*	*C. auriculatum*
*trnQ-psbK*-IGS_F	AAACCCGTTGCCTTACC	7558–7980	249	419
*trnQ-psbK*-IGS_R	AGATTGGAGTTGACAAATAACG			
*rps2-rpoC2*-IGS_F	GGTCTACCACTATAAACTAAAC	16,953–17,582	629	282
*rps2-rpoC2*-IGS_R	GCGGTGATACTCATATACA			
*psaJ-rpl33*-IGS_F	CACCGTTATTTCCTCCGTTGATA	74465–74806	342, 360	249, 250
*psaJ-rpl33*-IGS_R	CCTTACCGAGCATTTGCGA			

**Table 2:** Herbal medicine material from 10 commercial dried Baekshuoh products used in this study

Herbal Markets	Material Component	Source
A	Dried Radix of Baekshuoh products (300 g)	Yeongju-si, Gyeongsangbuk-do, Korea
B		Gangwon-do, Korea
C		Yeongju-si, Gyeongsangbuk-do, Korea
D		Yeongju-si, Gyeongsangbuk-do, Korea
E		Yeongju-si, Gyeongsangbuk-do, Korea
F		Bonghwa-gun, Gyeongsangbuk-do, Korea
G		Bonghwa-gun, Gyeongsangbuk-do, Korea
H		Yeongju-si, Gyeongsangbuk-do, Korea
I		Sancheong-gun, Gyeongsangnam-do, Korea
J		Yeongwol-gun, Gangwon-do, Korea

**Table 3:** Chloroplast sequences of *C. wilfordii* and *C. auriculatum* used in this study

No.	Species	Research Group	NCBI Accessions of Chloroplast Genome	Sequence Length (bp)
**1**	*C. wilfordii*	Lab. of Functional Crop Genomics & Biotechnology, Department of Plant Sciences College of Agriculture and Life Sciences, Seoul National University, Korea	NC029459	161,241
**2**			KT220733	
**3**	*C. auriculatum*	Lab. of Functional Crop Genomics & Biotechnology, Department of Plant Sciences College of Agriculture and Life Sciences, Seoul National University, Korea	NC029460	160,840
**4**			KT220734	
**5**		Department of Herbal Crop Research, National Institute of Horticultural and Herbal Science, Rural Development Administration, Eumseong, Korea	KU900231	160,203
